# Anion Exchanger 2 Regulates Dectin-1-Dependent Phagocytosis and Killing of *Candida albicans*

**DOI:** 10.1371/journal.pone.0158893

**Published:** 2016-07-08

**Authors:** Katia Urso, Julia F. Charles, Gary E. Shull, Antonios O. Aliprantis, Barbara Balestrieri

**Affiliations:** 1 Department of Medicine, Division of Rheumatology, Immunology and Allergy, Brigham and Women’s, Hospital and Harvard Medical School, Boston, Massachusetts, United States of America; 2 Department of Molecular Genetics, University of Cincinnati College of Medicine, Cincinnati, Ohio, United States of America; 3 Jeff and Penny Vinik Center for Allergic Disease Research, Division of Rheumatology, Immunology and Allergy, Brigham and Women’s Hospital, Harvard Medical School, Boston, Massachusetts, United States of America; University of Birmingham, UNITED KINGDOM

## Abstract

Anion exchanger 2 (Ae2; gene symbol, *Slc4a2*) is a plasma membrane Cl^-^/HCO_3_^-^ exchanger expressed in the gastrointestinal tract, kidney and bone. We have previously shown that Ae2 is required for the function of osteoclasts, bone resorbing cells of the macrophage lineage, to maintain homeostatic cytoplasmic pH and electroneutrality during acid secretion. Macrophages require endosomal acidification for pathogen killing during the process known as phagocytosis. Chloride is thought to be the principal ion responsible for maintaining electroneutrality during organelle acidification, but whether Cl^-^/HCO_3_^-^ exchangers such as Ae2 contribute to macrophage function is not known. In this study we investigated the role of Ae2 in primary macrophages during phagocytosis. We find that Ae2 is expressed in macrophages where it regulates intracellular pH and the binding of Zymosan, a fungal cell wall derivative. Surprisingly, the transcription and surface expression of *Dectin-1*, the major phagocytic receptor for *Candida albicans* (*C*. *albicans*) and Zymosan, is reduced in the absence of Ae2. As a consequence, Zymosan-induced *Tnfα* expression is also impaired in *Ae2-*deficient macrophages. Similar to *Ae2* deficiency, pharmacological alkalinization of lysosomal pH with bafilomycin A decreases both *Dectin-1* mRNA and cell surface expression. Finally, *Ae2-*deficient macrophages demonstrate defective phagocytosis and killing of the human pathogenic fungus *C*. *albicans*. Our results strongly suggest that Ae2 is a critical factor in the innate response to *C*. *albicans*. This study represents an important contribution to a better understanding of how *Dectin-1* expression and fungal clearance is regulated.

## Introduction

Ae2 is a Cl^-^/HCO3^-^ anion exchanger that belongs to the Slc4 (Solute Carrier Family 4) protein family together with Ae1 and Ae3. Ae2 is the most widely expressed Slc4a member and has been found in the gastrointestinal tract, kidney and bone [1]. *Ae2*^*-/-*^ mice have gastric achlorhydria, osteopetrosis and die soon after birth [2]. We have previously reported that Ae2 is expressed on the plasma membrane of osteoclasts and is required for their activity by preventing cytoplasmic alkalinization during bone resorption [2, 3].

Similar to osteoclasts, macrophages derive from myeloid hematopoietic progenitors and depend on pH regulation for certain functions. Macrophages are specialized in pathogen recognition and destruction. Bacterial and fungal pathogens are detected by pattern recognition receptors (PRRs) that recognize conserved structures of microorganisms, called pathogen-associated molecular patterns (PAMPs). After recognition and engagement they are engulfed into membrane-derived vesicles by a process known as phagocytosis. In the cytosol, phagocytic vesicles fuse with endosomes and finally with lysosomes to form phagolysosomes. During this process, the content of the vesicles becomes gradually more acidic (minimum pH = 4.5) through the activity of vacuolar proton pump H^+^-ATPase. Finally pH-dependent proteolytic enzymes are activated to kill and degrade the pathogen. This change in pH is critical for phagosome maturation, for killing of the pathogen and therefore for completion of the phagocytic process [4–7]. Anion movement is required to maintain electroneutrality during acidification, and defects in chloride transport disrupt endosomal acidification in a number of cell types [8, 9] but a role for Cl^-^/HCO_3_^-^ exchangers such as Ae2 in macrophage function has not been described previously.

The innate immune system is critical in the defense against fungal pathogens such as *C albicans*. Engulfment and killing by macrophages contributes to the innate host defense against fungal pathogens [10]. *C*. *albicans* is the predominant human pathogen among the *Candida* species [11] and is recognized by macrophages via cell wall β-glucans and mannosylated proteins. Mannosylated proteins are recognized by C-type lectins including Dectin-2, mannose receptor (MR), Galectin-3, and CD209 (SIGNR-1) [12–14]. β-glucans are recognized by the PRR C-type lectin receptor Dectin-1. Dectin-1 ligation by β-glucans induces both pathogen internalization and, together with Toll-like receptor 2 (TLR-2), cytokine production [15, 16]. Dectin-1 also amplifies TLR2 response through activation of the nuclear factor of activated T-cells (NFAT) pathway to augment pro-inflammatory cytokine production [17–19]. The fungal cell wall derivative Zymosan is similarly engaged by macrophages through the PRRs Dectin-1 and TLR2, inducing phagocytosis and a pro-inflammatory cytokine expression program [12, 20]. *Candida* infections (candidiasis) are a growing problem among hospitalized and immunocompromised patients. Despite the availability of antifungal treatments, these infections have high (40%) mortality rates and resistance to anti-fungals is an increasing issue [11]. Thus, a better understanding of anti-fungal host defense is imperative.

We sought to investigate the contribution of Ae2 to the process of fungal phagocytosis in macrophages. We found that absence of Ae2 expression increases cytosolic pH in primary macrophages. Additionally, *Ae2*-deficient macrophages are unable to phagocytose Zymosan particles. We demonstrate that this is likely a result of a specific requirement for Ae2 for optimal expression of the major receptor responsible of *C*. *albicans* binding, Dectin-1 [5]. We find that, *Ae2* expression is necessary for Dectin-1-dependent cytokine production, pathogen internalization and killing of *C*. *albicans*. This study contributes to a better understanding of pathways that regulate Dectin-1 expression and therefore the antifungal host defense by macrophages. Anion exchanger activity and intracellular pH maintenance become critical factors to take into account when designing new antifungal drugs to prevent fungal infections in hospitalized and immunocompromised patients.

## Methods

### Mice

**Fig 1:**
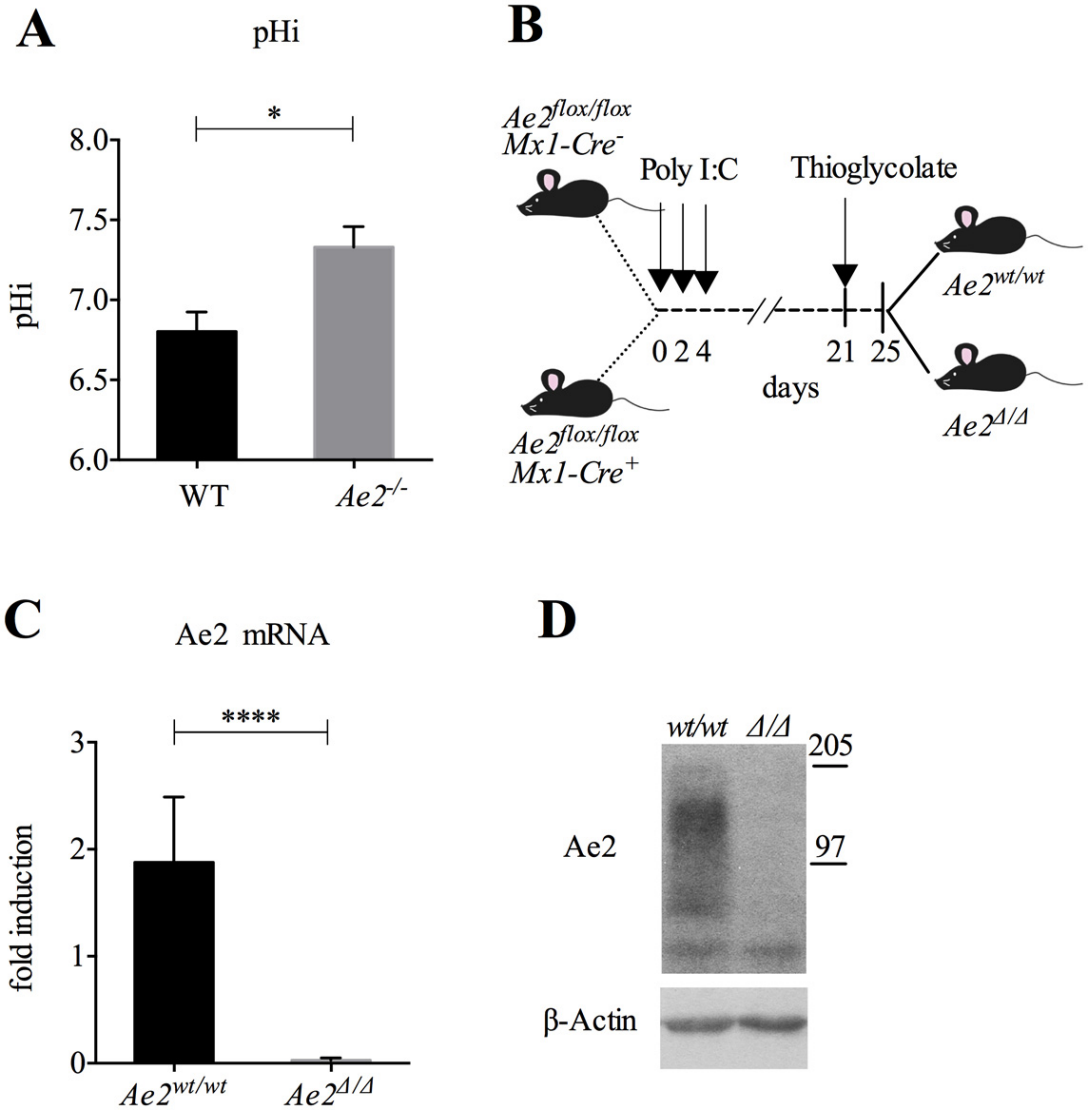
Ae2 is expressed in macrophages. (A) Intracellular pH (pHi) was measured in peritoneal macrophages isolated from 3 weeks old WT or *Ae2*-deficient mice (*Ae2*^*-/-*^). n = 3–4; *p<0.05, Mann-Whitney test. (B) Schematic representation of poly I:C (250 μg x 3) and thioglycollate treatment of *Ae2*^*flox/flox*^ *Mx1-Cre*^*-*^ and *Ae2*^*flox/flox*^ *Mx1-Cre*^*+*^ mice. (C, D) Expression of Ae2 in thioglycollate-elicited peritoneal macrophages isolated from *Ae2*^*wt/wt*^ and *Ae2*^*Δ/Δ*^ mice. Quantitative PCR showing *Ae2* RNA expression (n = 10 and 11; **** p<0.0001, Mann-Whitney test) (C) and western blot showing Ae2 protein levels (D).

*Ae2*-deficient mice (*Ae2*^*-/-*^) were originally described in [21]. *Ae2*^*flox/flox*^ *Mx1-Cre* mice on the C57/BL6 background were previously described in [3]. To induce expression of the *Cre* recombinase, mice were treated intraperitoneally (i.p) with poly I:C (250 mg) every other day for a total of 3 doses (hereafter referred to as *Ae2*^*Δ/Δ*^). Littermates without the *Mx1-Cre* transgene were identically treated with poly I:C (hereafter referred to as *Ae2*^*wt/wt*^*)*. Two weeks after the last poly I:C dose, mice received a single *i*.*p*. injection (1.5ml) of 3% thioglycollate solution (Difco) and were sacrificed 4 days later to collect peritoneal macrophages (Fig 1A). Wild-type C57/BL6 (WT) mice purchased from Jackson were used for pharmacological treatment experiments. All mice were housed in a specific pathogen-free animal facility at the Harvard School of Public Health. All the animals were sacrificed by asphyxiation with CO2 followed by cervical dislocation.

### Ethical approval

The study was approved by the Harvard Medical School Committee on Animals, IACUC protocol 04911. Mice were monitored for pain or distress and euthanized as specified by the humane endpoint criteria of the HMS IACUC.

### Cell isolation, culture and stimulation

Macrophages were isolated by washing the peritoneal cavity with ice-cold HBSS (Hank’s Balanced Salt Solution, Corning), washed and resuspended in complete media (RPMI 1640 (Corning CellGro), 10% Fetal Bovine Serum, 2 mM L-glutamine, 100 units/ml penicillin, and 100 μg/ml streptomycin, 10 mM Hepes, 50 μM β-Mercaptoethanol). 3.3x10^5^ cells were plated on sterile glass coverslips in 24-well tissue culture plates for phagocytosis experiments [22]. 2x10^6^ cells were seeded in 6 well tissue culture plates for all the other experiments. Cells were incubated with Bafilomycin A1 (25 nM, Sigma) for 6 and 16h.

### Synchronized *C*. *albicans* phagocytosis and killing assay

*C*. *albicans* (American Type Culture Collection 10231) was cultured as previously described [23]. Briefly, *C*. *albicans* was streaked on Sabouraud agar plates (BD, cat # 221849) and incubated at 37°C for 48 h, then kept at 4°C. The day before the experiment one colony was streaked on a fresh plate and incubated at 37°C. *C*. *albicans* were scraped from the plate, washed in endotoxin-free H_2_O (E-Toxate water, Sigma), and counted. Only colonies in the yeast form were used for experiments. After removing non-adherent cells, macrophages were incubated for another hour at 37°C. *C*. *albicans* (multiplicity of infection, MOI, of 5) were then pelleted (30 × *g*) onto peritoneal macrophages and incubated for the indicated time points at 37°C in 5% CO_2_. After washing to remove non-phagocytosed microorganisms, samples were either stained with a modified Wright Giemsa staining commercially available (Diff-Quick, Protocol HEMA 3 stain, Fisher Scientific Company LLC) to visualize macrophages [22, 23], for the phagocytosis assay, (15 min, 30 min, 1 h). Diff-Quick stained macrophages have a blue nucleus and a pink to purple cytoplasm [22, 23]. Alternatively macrophages were incubated with *C*. *albicans* for 15 min, washed and after 1 h and 3 h incubation to allow killing, cells were lysed in endotoxin-free H_2_O for the killing assay. Lysate dilutions were plated on Sabouraud agar plates and incubated for 24 h. The number of yeast colonies grown after 24 h (CFU, colony forming units) were counted manually [23]. Killing capacity of the cells was obtained by normalizing the CFU by the number of yeast cells internalized at the early time point, as counted in the corresponding sample stained with Diff-Quick after 15 min incubation [23]. The percentage of yeast killed by the macrophages was then determined as follows: for each mouse sample, the CFU number was divided by the phagocytic index at 15 min to account for the internalized *C*. *albicans* [23].

### Synchronized Zymosan phagocytosis and binding assay

After removing non-adherent cells, macrophages were incubated for another hour at 37°C. The cells were then washed once with cold complete media and cooled on ice for 5 min before the addition of Zymosan A (Sigma, 10 particles/cell, ppc) for 15 min on ice. The cells were then warmed by adding warm complete media and incubated at 37°C for the indicated period of time [22]. Zymosan particles surrounded by cell membrane were considered internalized. For binding assays, macrophages were preincubated for 30 min with 4 μM cytochalasin D (Sigma) and then stimulated with Zymosan at 10 ppc in complete media containing 4 μM cytochalasin D for 15 min and 30 min. Cells were washed extensively to remove unbound Zymosan, stained with Diff-Quik. In all of the experiments, non-opsonized Zymosan was used [22].

### Measurement of phagocytosis and binding

Diff-Quick staining and light microscopy was used to follow time-dependent *C*. *albicans* and Zymosan phagocytosis (Leica DM 2000, DFC3000 camera, Fluotar 40X objective). The phagocytic index was calculated by dividing the number of phagosomes by the total number of cells in a field, which was multiplied by the percentage of cells phagocytosing at least three particles or fungal cells [9, 22–24]. Similarly, the binding index was obtained by dividing the number of bound particles by the total number of cells in the field, multiplied by the percentage of cells binding at least three particles [23]. Pictures were acquired with Leica software applying the same contrast conditions among experiments.

### Measurement of cytosolic pH (intracellular pH, pHi)

Cytosolic pH was measured in peritoneal macrophages isolated from 3 week-old littermate WT and *Ae2*^*-/-*^ mice. Cells were immediately stained with pHrodo Red AM intracellular pH indicator (Molecular Probes). A pH standard curve, obtained by using the intracellular pH indicator buffer kit (Molecular Probes), was used to calculate the macrophage pHi according to the manufacturer’s instructions. Stained cells were analyzed with the FACS Canto II (BD) and Flowjo software version 10.6.

### RNA preparation and q-PCR

Macrophage RNA was extracted with Trizol reagent (Qiagen) following the manufacturers instructions. RNA was reverse transcribed into cDNA with the Affinity Script qPCR cDNA Synthesis Kit (Agilent Technology). Real-time quantitative PCR (q-PCR) was performed using Sybr green reagent (Life Technologies). Primers are listed in Table 1. Data were normalized to *Hprt (Hypoxanthine-guanine phosporibosyltransferase)* and presented as relative quantification calculated by the conventional 2^-ΔCt^ method as described in [6, 25, 26].

**Table 1:** List of primers.

*Gene*	Forward	Reverse
*Ae2* exon 8^a^	CCCATGAGGTGTTTGTGG	TCCACATCCTCCTCGAATTT
*Cd36*^b^	AGATGACGTGGCAAAGAACAG	CCTTGGCTAGATAACGAACTCTG
*Dectin-1*^b^	ATGGTTCTGGGAGGATGGAT	GCTTTCCTGGGGAGCTGTAT
*Hprt*^a^	GTTAAGCAGTACAGCCCCAAA	AGGGCATATCCAACAACAAACTT
*Dectin-2*	GACCAGCCCAGTAGAAGACTA	GGCAGCATCCCCACATTTTT
*Marco*^b^	CCTCCAGGGACTTACGGGT	CCAGTGAGACCTATGTCACCT
*SrI-II*^b^	TTCACTGGATGCAATCTCCAAG	CTGGACTTCTGCTGATACTTTGT
*Tnfα*^b^	CATCTTCTCAAAATTCGAGTGACAA	TGGGAGTAGACAAGGTACAACCC

^*a*^ Primers were described in [3].

^*b*^ primers were designed with Primer Blast website tool.

### Western Blot

Cells were washed with ice-cold HBSS and lysed with Laemmli buffer and boiled for 5 min. Total cell extracts were separated by SDS-PAGE and transferred to nitrocellulose membranes, which were then incubated over-night at 4°C with the primary antibodies as follows anti-Ae2 [27] (0.4 μg/ml), anti-β-Actin (33 ng/ml, Cell Signaling). Membrane-bound antibody was detected with enhanced chemiluminescence (ECL) detection reagent (GE Healthcare Lifesciences).

### Flow cytometry

Macrophages were gently detached from the plate by 5 min incubation at 37°C with 5 mM EDTA HBSS. Single cell suspensions were stained for 30 minutes at 4°C with the following fluorochrome-associated antibodies in the presence of Fc-block (Biolegend): αDectin-1 (4 μg/ml, Serotec), αCD64 (4 μg/ml, Biolegend). Live/dead staining was performed using the 7-AAD staining solution (Biolegend). Stained cells were analyzed with the FACS Canto II (BD) and Flowjo software version 10.6.

### Statistical Analysis

Statistical analysis and normalization methods were selected with the help of the Harvard Catalyst Biostatistical Consulting Service. Experiments with 2 groups were analyzed with non–parametric Mann-Whitney test. Two-Way ANOVA followed by Bonferroni post-test correction for multiple hypothesis testing was used for experiments with multiple groups. A significance level α = 0.05 was considered significant. All statistical analyses were performed with GraphPad Prism version 6. Since we detected considerable variability among independent experiments, individual data points of each experiment have been divided by the internal experimental mean to normalize inter-assay variation. Error bars represent standard deviation (SD). All the experiments were performed at least three times unless otherwise specified. Harvard Catalyst Biostatistical Consulting Service is supported from Harvard Catalyst/The Harvard Clinical and Translational Science Center (National Center for Research Resources and the National Center for Advancing Translational Sciences, National Institutes of Health Award UL1 TR001102) and financial contributions from Harvard University and its affiliated academic healthcare centers. The scientific content is solely the responsibility of the authors and does not necessarily represent the official views of Harvard Catalyst, Harvard University and its affiliated academic healthcare centers, or the National Institutes of Health.

## Results

### Expression of Ae2 in macrophages is required for pHi regulation

We have previously shown that Ae2 is highly expressed in mature osteoclasts after differentiation from myeloid precursors [3]. The purpose of this study was to evaluate the expression and function of this anion transporter in macrophages, that, similar to osteoclasts, belong to the myeloid lineage. Because Ae2 has a role in the maintenance of pHi in several cell types, including osteoclasts [3, 27, 28], we sought to determine if Ae2 was also required for maintenance of pHi in macrophages. We isolated peritoneal macrophages from *Ae2*-defient mice (*Ae2*^*-/-*^*)* and found that, compared to macrophages isolated from wild-type (WT) littermates, *Ae2*^*-/-*^ macrophages had a significantly higher pHi (Fig 1A). Since germ line deletion of *Ae2* results in early lethality (20–21 days), to further investigate the role of Ae2 in macrophages we used the inducible *Ae2*^*fl/fl*^ *Mx1-Cre* mouse strain (*Ae2*^*Δ/Δ*^*)* [3] (Fig 1B), in which *Ae2* is broadly deleted post-natally, including in hematopoietic cells. First, we analyzed the expression of *Ae2* in thioglycollate-elicited peritoneal macrophages at the mRNA and protein levels. *Ae2* transcript was present in macrophages as assessed by qPCR (Fig 1C), while protein analysis by western blot showed the expression of multiple isoforms of Ae2 (Fig 1D), as reported for other cell types [27, 29, 30]. However, *Ae2* expression was undetectable in macrophages isolated from *Ae2*^Δ/Δ^ mice (Fig 1C and 1D), demonstrating efficient induced deletion of the gene in poly I:C treated *Ae2*^*fl/fl*^ *Mx1-Cre* macrophages, as previously reported in osteoclasts [3].

### Ae2 is required for the binding and internalization of Zymosan

**Fig 2:**
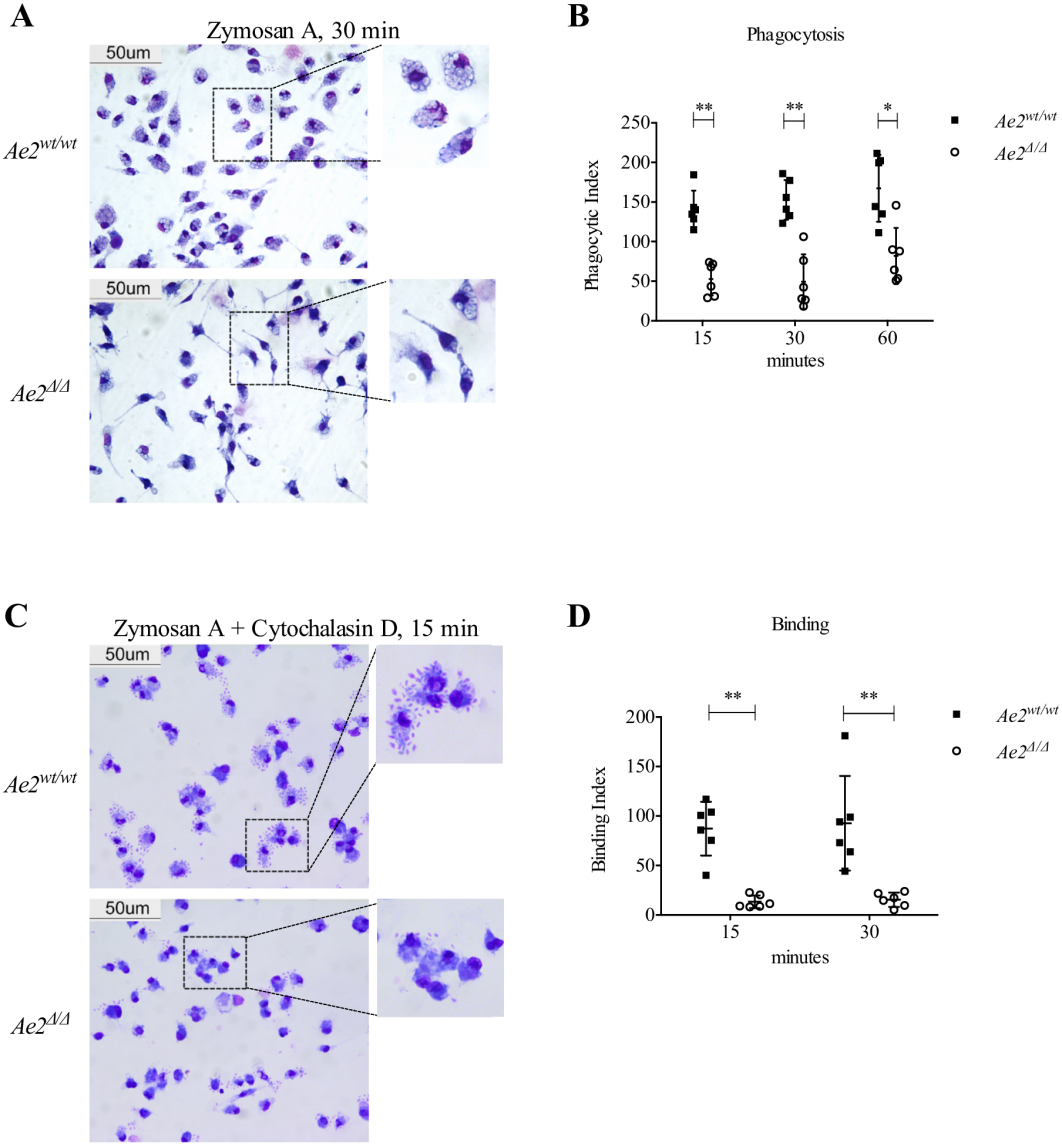
Phagocytosis of Zymosan by macrophages requires Ae2. *Ae2*^*wt/wt*^ and *Ae2*^*Δ/Δ*^ macrophages were incubated with Zymosan (5 ppc) for the indicated time in the presence or absence of cytochalasin D, stained with Diff-Quick, and phagocytosis and binding were quantitated as described in the methods. (A) Representative images obtained after 30 min incubation with Zymosan, scale bar 50 μm. 2X digitally enlarged details are also shown. (B) Phagocytic index for *Ae2*^*wt/wt*^ and *Ae2*^*Δ/Δ*^ macrophages at the indicated time points after Zymosan addition. (n = 6; * p<0.05 ** p<0.01, Mann-Whitney test). (C) Representative images and 2X digitally enlarged detail of *Ae2*^*wt/wt*^ and *Ae2*^*Δ/Δ*^ macrophages treated with cytochalasin D (4 μM) for 30 min prior to incubation with Zymosan particles (5 ppc). (D) Binding index of Zymosan to *Ae2*^*wt/wt*^ and *Ae2*^*Δ/Δ*^ macrophages at the indicated time points (n = 6; ** p<0.01, Mann-Whitney test).

We have previously demonstrated that Ae2 is required for pHi regulation in osteoclasts and, as a consequence, for bone resorption [3]. Macrophage phagocytosis is also a pH-dependent process [4]. Because macrophages lacking Ae2 have a significantly higher pHi compared to WT macrophages (Fig 1A), we postulated that Ae2 would be required for phagocytosis by macrophages. To test this hypothesis, we performed a phagocytosis assay using Zymosan, a fungal wall derivative. Time-dependent internalization of Zymosan particles by *Ae2*^*wt/wt*^ and *Ae2*^*Δ/Δ*^ macrophages showed that *Ae2*^*Δ/Δ*^ macrophages were significantly less efficient than control cells in internalizing Zymosan particles at any time point analyzed (Fig 2A and 2B). Since the defect was observed at the earliest time point (15 min), we reasoned that this phenotype could reflect a defect in any of the initial steps of phagocytosis, including binding and uptake. To ascertain the binding ability of *Ae2*^*Δ/Δ*^ macrophages, cells were stimulated with Zymosan in the presence of cytochalasin D, a drug that prevents actin polymerization and hence internalization. Our results showed that *Ae2*^*Δ/Δ*^ macrophages bound significantly fewer Zymosan particles when compared to *Ae2*^*wt/wt*^ controls (Fig 2C and 2D). The reduced number of particles bound on the macrophages surface in *Ae2*^*Δ/Δ*^ macrophages indicated a defect in binding of fungal wall components.

### Tnfα induction by Zymosan is impaired in Ae2^Δ/Δ^ macrophages

**Fig 3:**
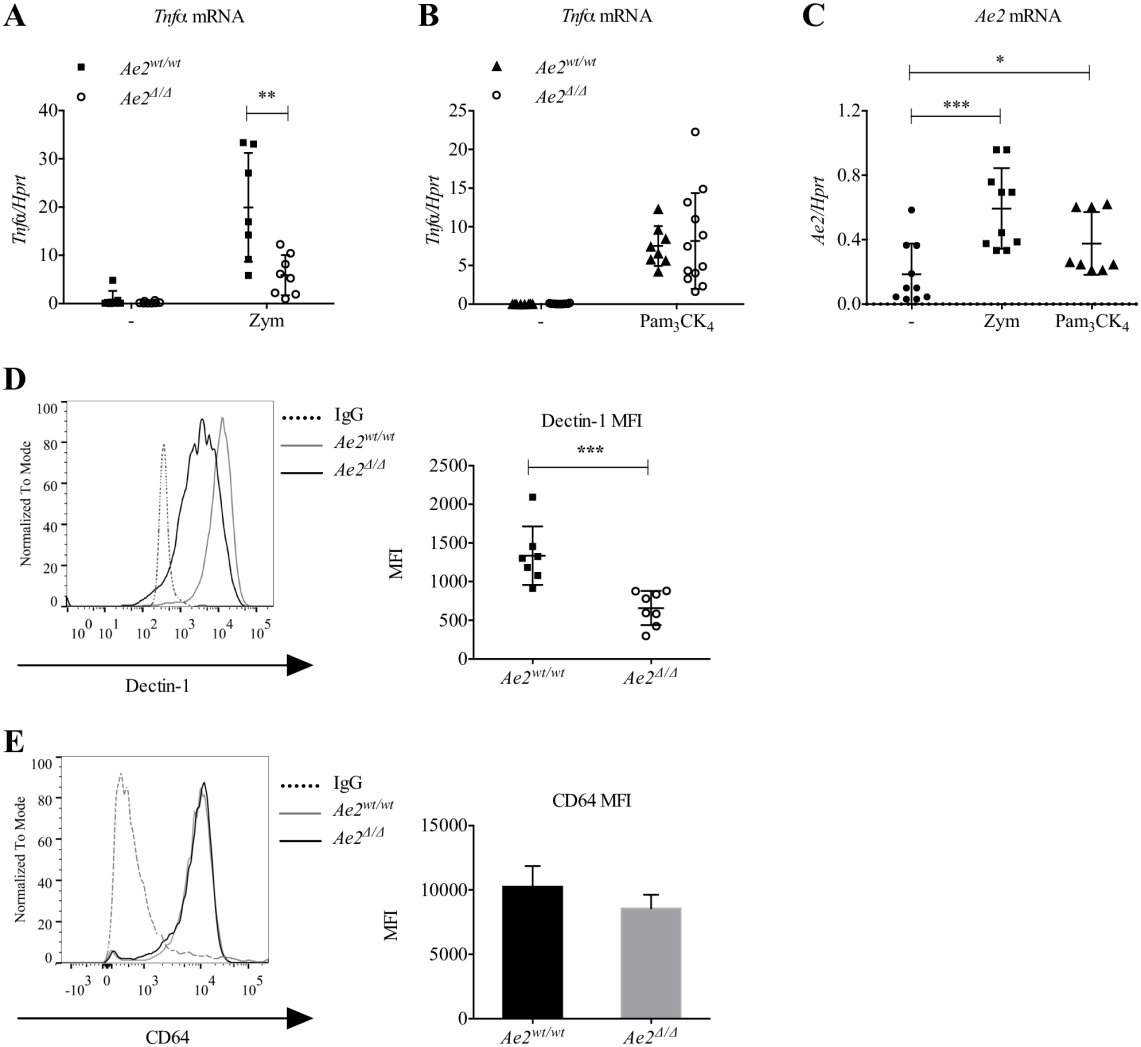
Dectin-1 response and expression is Ae2-dependent. (A and B) *Tnfα* mRNA expression relative to *Hprt* in *Ae2*^*wt/wt*^ and *Ae2*^*Δ/Δ*^ macrophages stimulated for 6h with Zymosan (5 ppc) (A) or PAM_3_CK_4_ (B) (n = 8–10, ** p<0.01, Mann-Whitney test). (C) *Ae2* mRNA expression relative to *Hprt by* WT macrophages stimulated for 6h with Zymosan (Zym, 5 ppc) or PAM_3_CK_4_ (100 ng/ml) (n = 8–12, * p<0.05, *** p<0.001, Mann-Whitney test). (D) Representative flow cytometry histogram with quantification by mean fluorescence intensity (MFI) of Dectin-1 expression on cell surface of *Ae2*^*wt/wt*^ *and Ae2*^*Δ/Δ*^ macrophages (n = 8, *** p<0.001, Mann-Whitney test). (E) Representative flow cytometry histogram and MFI of CD64 expression on cell surface of *Ae2*^*wt/wt*^ and *Ae2* ^*Δ/Δ*^ macrophages (n = 5).

The data in Fig 2 suggested that Ae2 is required for Zymosan binding. Zymosan is recognized and engaged through two receptors, Dectin-1 and TLR2 [15–19]. Zymosan binding to either receptor induces expression of inflammatory cytokines, including *Tnfα*. In order to determine if *Tnfα* mRNA expression was compromised in *Ae2*^*Δ/Δ*^ macrophages, we stimulated the cells with either Zymosan, which activates both receptors, or PAM_3_CK_4_, a molecule that specifically activates TLR2 [31] but not Dectin-1. As expected, Zymosan stimulation upregulated the transcription of *Tnfα* in *Ae2*^*wt/wt*^ macrophages. In contrast, *Tnfα* mRNA expression was significantly impaired in *Ae2*^*Δ/Δ*^ macrophages compared to equally treated *Ae2*^*wt/wt*^ controls (Fig 3A). However *Tnfα* was similarly induced by PAM_3_CK_4_ in macrophages of both genotypes indicating that *Ae2*^*Δ/Δ*^ macrophages did not have a global defect in *Tnfα* transcription but rather that the Dectin-1 specific pathway was compromised (Fig 3B). Interestingly, we also found that Zymosan and, to a lesser extent PAM_3_CK_4_, induced the transcription of *Ae2* (Fig 3C) suggesting a positive feedback mechanism following macrophage activation. These data indicated that Dectin-1, and not TLR2, was the Zymosan receptor affected by *Ae2*-deficiency.

### Ae2 is required for Dectin-1 expression

Since Dectin-1-dependent binding and *Tnfα* expression were impaired in *Ae2*^*Δ/Δ*^ macrophages, we postulated that the expression of Dectin-1 on the surface of macrophages would be reduced in the absence of Ae2. Indeed, flow cytometric analysis showed that cell surface Dectin-1 on resting *Ae2*^*Δ/Δ*^ macrophages was significantly reduced compared to control cells (Fig 3D). To ascertain whether other major macrophage receptors were affected by the absence of Ae2, we stained macrophages for CD64, the FcγR1 receptor [32]. The expression of CD64 was similar in macrophages of both genotypes (Fig 3E). These data suggested that Ae2 might have a selective effect on Dectin-1 expression in resting macrophages.

**Fig 4:**
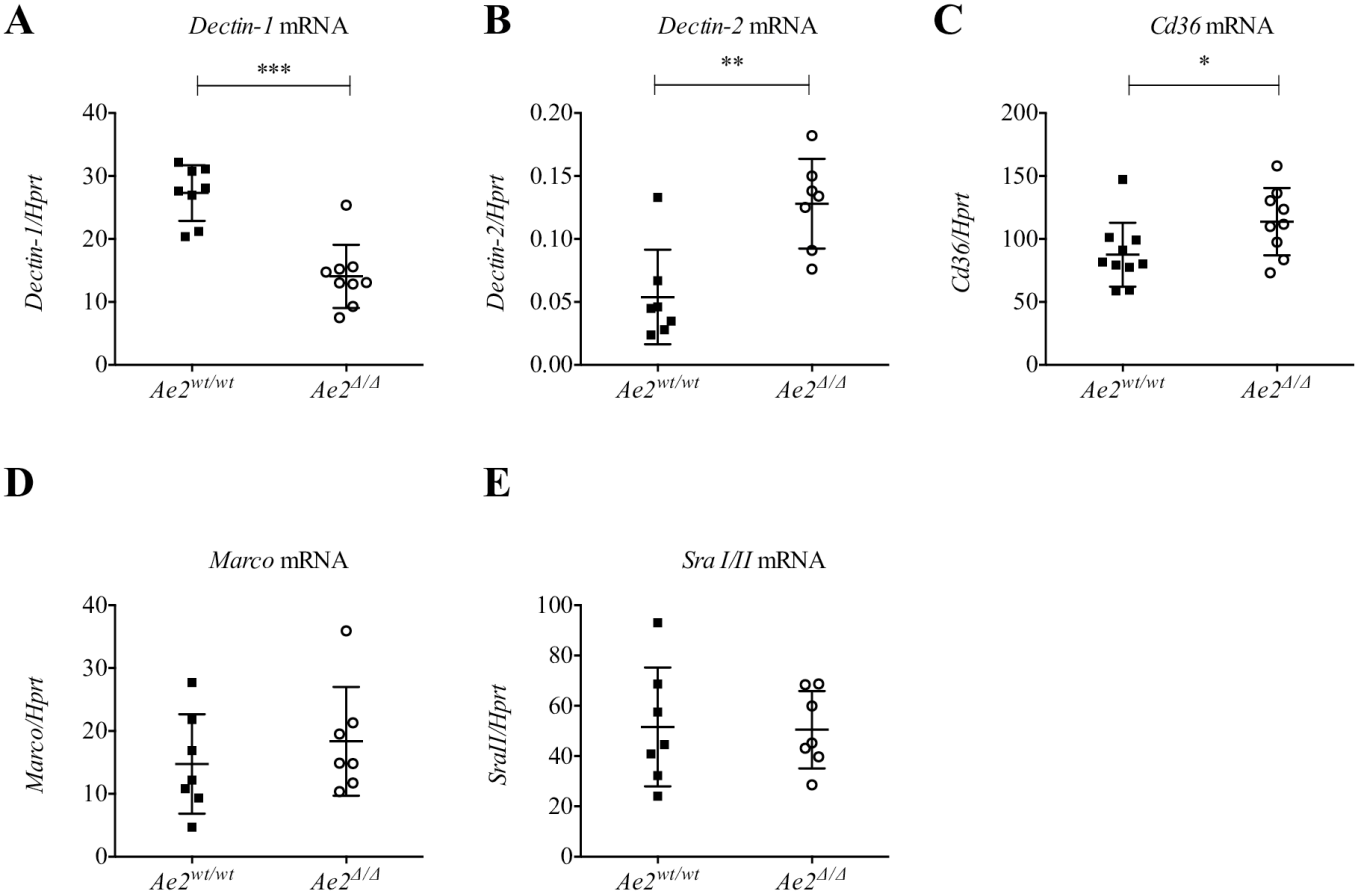
*Dectin-1* gene expression in macrophages requires Ae2. Gene expression relative to *Hprt* for (A) *Dectin-1*, (B) *Dectin-2*, (C) *Cd36*, (D) *Marco*, and (E) *SraI-II*, for *Ae2*^*wt/wt*^ compared to *Ae2*^*Δ/Δ*^ macrophages as determined by qPCR (n = 7–10, * p<0.05, ** p<0.01, *** p<0.001, Mann-Whitney test).

We reasoned that the reduced Dectin-1 cell surface expression in *Ae2*^*Δ/Δ*^ macrophages could be due to reduced gene transcription. Therefore we analyzed *Dectin-1* expression by qPCR. Interestingly, macrophages lacking Ae2 displayed reduced *Dectin-1* transcripts (Fig 4A). These data suggested that Ae2 controls *Dectin-1* gene transcription or mRNA stability either directly or indirectly. In contrast, the mRNA expression of *Dectin-2*, a receptor involved in phagocytosis of *C*. *albicans* hyphae through α-mannans [19, 25, 26, 33, 34] and *Cd36*, a scavenger receptor that participates in *C*. *albicans* recognition via β-glucans [35], were significantly upregulated in absence of Ae2 (Fig 4B and 4C). The transcription of other scavenger receptors, *SraI/II* and *Marco*, was unchanged (Fig 4D and 4E).

### Pharmacological alteration of pH inhibits Dectin-1 expression

**Fig 5:**
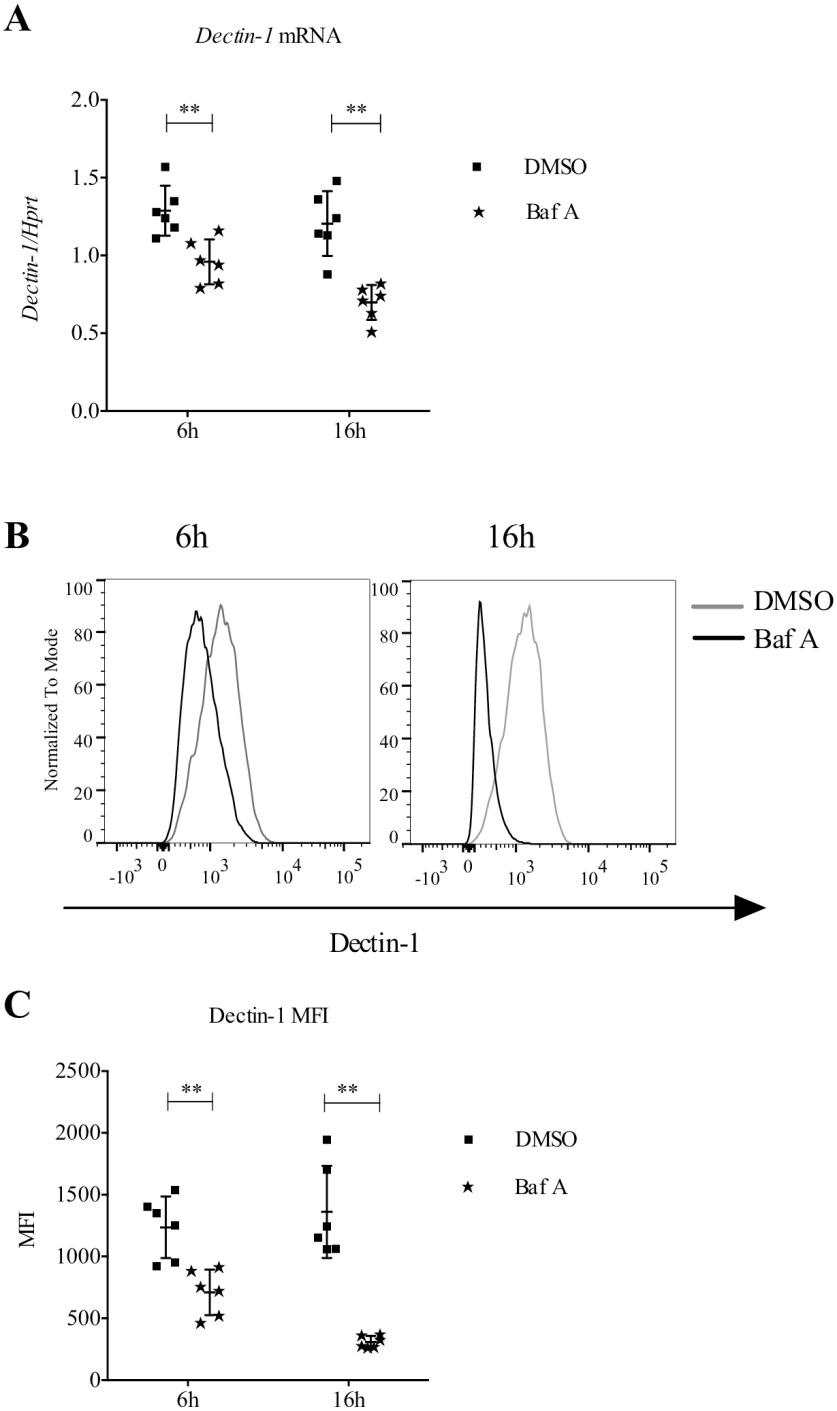
Pharmacological alkalinization of pHi inhibits *Dectin-1* expression. (A) *Dectin-1* expression relative to *Hprt* by wild-type macrophages treated with bafilomycin A (Baf A, 25 nM) or DMSO control for 6 and 16 h. (B) Representative flow cytometry histogram and (C) quantification by MFI showing decreased Dectin-1 in wild-type macrophages treated with Baf A for 6 and 16 h compared to DMSO control (n = 6, ** p<0.01, Mann-Whitney test).

*Ae2*^-/-^ osteoclasts have a more alkaline pH in both their cytosol and lysosomes, due to the absence of the anion exchanger activity [3]. We hypothesized that, similarly to osteoclasts, macrophages lacking Ae2 have also a defect in lysosomal pH and this alteration interferes with *Dectin-1* expression. To test this hypothesis WT macrophages were treated with bafilomycin A, an inhibitor of the vacuolar-type H+-ATPase. Inhibition of this enzyme blocks acidification of lysosomes but likely does not have a direct effect on anion exchange. *Dectin-1* mRNA and protein levels were analyzed in WT macrophages treated with bafilomycin A for 6 and 16 h. Bafilomycin A treatment strongly reduced *Dectin-1* mRNA (Fig 5A). Dectin-1 cell surface expression was also significantly reduced at both time points (Fig 5B and 5C), suggesting that lysosomal pH is a critical modulator of *Dectin-1* expression and subsequent activation of the phagocytosis machinery.

### Ae2 expression is required to phagocytose and kill *C*. *albicans*

**Fig 6:**
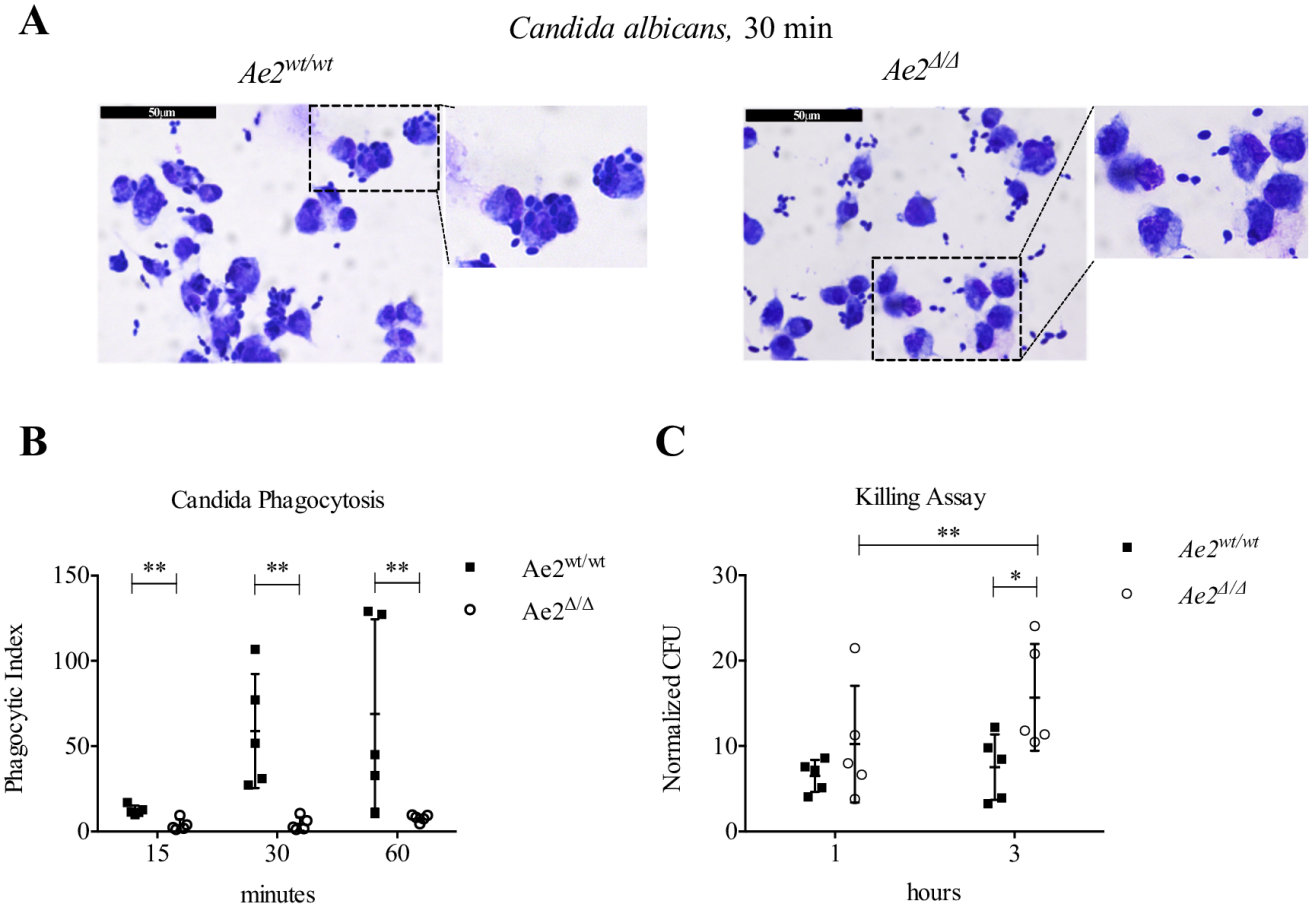
Ae2 is required for *C*. *albicans* phagocytosis and killing. (A) Representative images and 2X digitally enlarged detail of Diff-Quick stained *Ae2*^*wt/wt*^ and *Ae2*^*Δ/Δ*^ macrophages incubated with live *C*. *Albicans* (MOI 2) for 30 min, scale bar 50 μm. (B) Phagocytic index of *Ae2*^*wt/wt*^ and *Ae2*^*Δ/Δ*^ macrophages incubated with live *C*. *albicans* at the indicated time points (n = 6; ** p<0.01, Mann-Whitney test). (C) CFU (colony forming unit) from cell lysates of *Ae2*^*wt/wt*^ and *Ae2*^*Δ/Δ*^ macrophage incubated for the indicated time with live *C*. *albicans* were determined and normalized to the phagocytic index at 15 min (n = 5; * p<0.05, ** p<0.01, two-way ANOVA with Bonferroni post-test).

Our data indicate that Ae2 is required for Zymosan binding by promoting the expression of *Dectin-1* and that the transcription of this receptor is very sensitive to pH regulation. In addition, the bafilomycin experiments indicate that alkalinization of lysosomes in resting macrophages mimics the phenotype of *Ae2*^*Δ/Δ*^ macrophages by inhibiting *Dectin-1* expression. Pathogen killing is a process that is strongly dependent on lysosome acidification [4]. Thus, we reasoned that if the lack of the anion exchanger activity in macrophages is also altering lysosomal pH, pathogen killing might be affected. To test this hypothesis we performed a phagocytosis and killing assay using *C*. *albicans*. Similar to the Zymosan experiments shown in Fig 2, *Ae2*^*Δ/Δ*^ macrophages internalized less *C*. *albicans* than controls at all time points analyzed (Fig 6A and 6B). In addition, killing of engulfed *C*. *albicans* was impaired in *Ae2*^*Δ/Δ*^ macrophages, as assessed by our killing assay (Fig 6C) where the amount of live *C*. *albicans* recovered from *Ae2*^*Δ/Δ*^ macrophages after 3h of incubation was significantly higher than CFU from wild type cells. In addition, the increase in CFU recovered after 3h when compared to 1h of incubation (3 h *Ae2*^*Δ/Δ*^ *versus* 1 h *Ae2*^*Δ/Δ*^) also suggests that *C*. *albicans* was able to proliferate in *Ae2*^*Δ/Δ*^, but not in *Ae2*^*wt/wt*^, macrophages. Altogether, these data suggest that Ae2 in macrophages participates in two steps of fungal clearance: binding, by regulating *Dectin-1* expression, and final killing of the pathogen.

## Discussion

The SLC4A Cl^-^/HC03^-^ exchangers are well known regulators of intracellular pH and Cl^-^ trafficking [1]. In particular, a role for Ae2 in regulating pHi has been described in different cell types including osteoclasts [2, 3], CD8-T cells [28] and colon epithelial cells [27], but its expression and role in macrophages was completely unexplored. Here we show for the first time that *Ae2*^*-/-*^ macrophages have a more alkaline cytoplasm (Fig 1A), suggesting that Ae2 is required for pHi regulation in macrophages. This result is in line with our previous data showing that *Ae2*^*-/-*^ osteoclasts have a more alkaline pHi and consequently impaired bone resorbing function. [3]. Since *Ae2*^*-/-*^ mice die shortly after birth, we utilized an inducible mouse model (*Mx1-Cre*), which deletes Ae2 in hematopoietic cells, for the majority of our experiments, as we have previously done for studies of Ae2 function in osteoclasts [3]. Using *Ae2*^*flox/flox*^ *Mx1-Cre* mice, we show for the first time that Ae2 is present in WT macrophages (Fig 1C and 1D). Western blot analysis revealed that different isoforms of the protein are expressed in macrophages (Fig 1D), likely corresponding to the transcript variants derived from alternative promoters, as described in other tissues [27, 36, 37].

The use of the poly I:C induced *Mx1-Cre* system has been previously described by our and other groups [10, 38–42]. Poly I:C has an extremely short half-life *in vivo* [39, 43], thus systemic effects of poly I:C would not be expected to be durable [16]. This issue has been already examined experimentally, and no systemic effect of poly I:C was found 2 days after treatment (with the exception of modest effects on the thymus) [43]. In addition, 4 days after poly I:C or saline treatment, thioglycolate-elicited macrophages isolated from *Mx1-Cre*^*-*^ mice did not show differences in macrophage markers such as CD36 and PPARγ [42]. Thus the interval of 18 days between treatment and analysis followed in our protocol (Fig 1A) minimizes the likelihood that poly I:C directly affected the assays performed. In addition both groups of mice (*Cre*^*+*^ and *Cre*^*-*^) received the treatment. Thus, any effect of poly I:C treatment on myeloid lineages would equally affect both genotypes, therefore the differences observed are attributable to *Ae2*-deficiency.

As phagocytosis is a major function of macrophages, and the phagocytic process is known to be dependent on pH [4–7], we sought to investigate the role of Ae2 in this process. We found that Ae2 is required for binding and uptake of Zymosan by macrophages (Fig 2). Taking advantage of the ability of Zymosan particles to trigger pro-inflammatory cytokine expression, we also found that *Tnfα* was reduced in *Ae2*^*Δ/Δ*^ macrophages compared to controls (Fig 3A), but this defect was not observed when the cells were stimulated with the TLR2-specific ligand Pam3CK4 (Fig 3B). These data indicate that Ae2 may be specifically required for Dectin-1-dependent responses and highlight that, since Ae2 participates in the very early events of pathogen recognition, this transporter is also important for the downstream cytokine production.

We found that Dectin-1 protein and mRNA were specifically and significantly reduced in *Ae2*^*Δ/Δ*^ macrophages compared to controls (Figs 3D and 4A). Thus, the impaired response of *Ae2*^*Δ/Δ*^ macrophages to Zymosan is likely due to reduced Dectin-1 expression. Interestingly, the mRNA levels of *Dectin-2* and *Cd36* were increased, indicating a potential compensatory effect in the cells lacking Ae2 and Dectin-1 (Fig 4D and 4E).

Other studies have associated Ae2 with transcriptional regulation. In liver, cholangiocytes, the bile duct epithelia cells have an altered expression profile of genes related to oxidative stress and antigen presentation in the absence of Ae2 [10]. *Ae2*-deficiency in CD8^+^ T cells disrupts pHi and upregulates the transcription of both the cytokine IL-2 and its receptor IL2-R, resulting in uncontrolled proliferation after CD3 stimulation [28]. Similarly, our data show that *Ae2*-deficiency decreases *Dectin-1* mRNA in macrophages (Fig 4). Although the mechanism behind the reduction in the gene expression needs further investigation, this study contributes to a better understanding of *Dectin-1* regulation by indicating that Ae2 and pHi are critical factors.

We speculate that the *Ae2*-deficiency impairs *Dectin-1* expression and phagocytic function of macrophages via pHi, including cytosolic and lysosomal alkalinization. Indeed, we found that, similar to *Ae2*-deficiency (Fig 1A), treatment of resting macrophages with bafilomycin A, which alkalinizes lysosomes, also impairs *Dectin-1* transcription (Fig 5A) and Dectin-1 cell surface expression (Fig 5B and 5C). This decrease in surface expression of Dectin-1 is in line with previous studies where bafilomycin A treatment of RAW cells during phagocytosis of β-glucan beads caused retention of Dectin-1 on the endophagosome membrane and, as a consequence, impaired phagosome maturation and recruitment of TLR9, a receptor necessary to recognize the pathogenic DNA and further activate the cells [38, 44]. Although short-term pretreatment (30 min) with bafilomycin A reportedly did not inhibit phagocytosis [44], we found that longer treatment (6 and 16 hours) impairs Dectin-1 mRNA and protein expression on the cell surface (Fig 5B and 5C) and therefore could inhibit Zymosan or *C*. *albicans* binding. It is possible that pHi alkalinization caused by the *Ae2*-deficiency results in Dectin-1 retention on the endophagosome membrane, thus blocking phagosome maturation and potentially producing the defect in killing *C*. *albicans* seen in Fig 6C. Finally, we also found that Zymosan induces *Ae2* transcription (Fig 3C). This suggests that Ae2 is part of a positive feedback loop in macrophages, whereby exposure to fungal wall components increases the expression of Ae2, which further readies the cell for phagocytosis by augmenting Dectin-1 cell surface expression.

Dectin-1 is one the most important components of host defense against fungal pathogens, of which *C*. *albicans* is one of the most clinically important, resulting in 50,000 deaths per year in USA [11]. Polymorphisms in *Dectin-1* have been linked to susceptibility to fungal infections in humans [45]. Previous studies evaluating the relevance of Dectin-1 in *C*. *albicans* clearance *in vivo* indicated that mice of different strains are differentially susceptible to systemic fungal infection [16]. However, other reports have shown that Dectin-1 is required for β-glucans and Zymosan induced cell activation [15, 46, 47]. Thus, defining the pathways necessary to preserve optimal expression of this receptor is an important step forward for the improvement of candidiasis prevention and treatment. Interestingly, the use of small molecules to facilitate Cl^-^/HCO3^-^ antiport is currently under development and constitutes a promising way to enhance this pathway [48, 49]. Our data shed light on one pathway by which facilitation of Cl^-^/HCO3^-^ exchange might be beneficial in the host response to fungal infection.

## References

[1] Alper SL. Molecular physiology and genetics of Na+-independent SLC4 anion exchangers. The Journal of experimental biology. 2009;212(Pt 11):1672–83. 10.1242/jeb.029454 19448077 PMC2683012

[2] Wu J, Glimcher LH, Aliprantis AO. HCO3-/Cl- anion exchanger SLC4A2 is required for proper osteoclast differentiation and function. Proceedings of the National Academy of Sciences of the United States of America. 2008;105(44):16934–9. 10.1073/pnas.0808763105 18971331 PMC2579356

[3] Coury F, Zenger S, Stewart AK, Stephens S, Neff L, Tsang K, et al SLC4A2-mediated Cl-/HCO3- exchange activity is essential for calpain-dependent regulation of the actin cytoskeleton in osteoclasts. Proceedings of the National Academy of Sciences of the United States of America. 2013;110(6):2163–8. 10.1073/pnas.1206392110 23341620 PMC3568349

[4] Flannagan RS, Jaumouille V, Grinstein S. The cell biology of phagocytosis. Annual review of pathology. 2012;7:61–98. 10.1146/annurev-pathol-011811-132445 . 21910624

[5] Underhill DM, Goodridge HS. Information processing during phagocytosis. Nature reviews Immunology. 2012;12(7):492–502. 10.1038/nri3244 . 22699831 PMC5570470

[6] Gordon AH, Hart PD, Young MR. Ammonia inhibits phagosome-lysosome fusion in macrophages. Nature. 1980;286(5768):79–80. . 6993961 10.1038/286079a0

[7] Stuart LM, Ezekowitz RA. Phagocytosis: elegant complexity. Immunity. 2005;22(5):539–50. 10.1016/j.immuni.2005.05.002 . 15894272

[8] Nilius B, Droogmans G. Amazing chloride channels: an overview. Acta physiologica Scandinavica. 2003;177(2):119–47. 10.1046/j.1365-201X.2003.01060.x . 12558550

[9] Girotti M, Evans JH, Burke D, Leslie CC. Cytosolic phospholipase A2 translocates to forming phagosomes during phagocytosis of zymosan in macrophages. The Journal of biological chemistry. 2004;279(18):19113–21. 10.1074/jbc.M313867200 . 14963030

[10] Gilbert AS, Wheeler RT, May RC. Fungal Pathogens: Survival and Replication within Macrophages. Cold Spring Harbor perspectives in medicine. 2015;5(7):a019661 10.1101/cshperspect.a019661 . 25384769 PMC4484954

[11] Kullberg BJ, Arendrup MC. Invasive Candidiasis. The New England journal of medicine. 2015;373(15):1445–56. 10.1056/NEJMra1315399 . 26444731

[12] Gantner BN, Simmons RM, Canavera SJ, Akira S, Underhill DM. Collaborative induction of inflammatory responses by dectin-1 and Toll-like receptor 2. The Journal of experimental medicine. 2003;197(9):1107–17. 10.1084/jem.20021787 12719479 PMC2193968

[13] Netea MG, Gow NA, Munro CA, Bates S, Collins C, Ferwerda G, et al Immune sensing of Candida albicans requires cooperative recognition of mannans and glucans by lectin and Toll-like receptors. The Journal of clinical investigation. 2006;116(6):1642–50. 10.1172/JCI27114 16710478 PMC1462942

[14] Saijo S, Iwakura Y. Dectin-1 and Dectin-2 in innate immunity against fungi. International immunology. 2011;23(8):467–72. 10.1093/intimm/dxr046 . 21677049

[15] Brown GD, Gordon S. Immune recognition. A new receptor for beta-glucans. Nature. 2001;413(6851):36–7. 10.1038/35092620 . 11544516

[16] Naumann K, Wehner R, Schwarze A, Petzold C, Schmitz M, Rohayem J. Activation of dendritic cells by the novel Toll-like receptor 3 agonist RGC100. Clinical & developmental immunology. 2013;2013:283649. 10.1155/2013/283649 24454470 PMC3878805

[17] Greenblatt MB, Aliprantis A, Hu B, Glimcher LH. Calcineurin regulates innate antifungal immunity in neutrophils. The Journal of experimental medicine. 2010;207(5):923–31. 10.1084/jem.20092531 20421389 PMC2867274

[18] Netea MG, Joosten LA, van der Meer JW, Kullberg BJ, van de Veerdonk FL. Immune defence against Candida fungal infections. Nature reviews Immunology. 2015;15(10):630–42. 10.1038/nri3897 . 26388329

[19] Saijo S, Ikeda S, Yamabe K, Kakuta S, Ishigame H, Akitsu A, et al Dectin-2 recognition of alpha-mannans and induction of Th17 cell differentiation is essential for host defense against Candida albicans. Immunity. 2010;32(5):681–91. 10.1016/j.immuni.2010.05.001 . 20493731

[20] Sato M, Sano H, Iwaki D, Kudo K, Konishi M, Takahashi H, et al Direct binding of Toll-like receptor 2 to zymosan, and zymosan-induced NF-kappa B activation and TNF-alpha secretion are down-regulated by lung collectin surfactant protein A. Journal of immunology. 2003;171(1):417–25. . 12817025 10.4049/jimmunol.171.1.417

[21] Gawenis LR, Ledoussal C, Judd LM, Prasad V, Alper SL, Stuart-Tilley A, et al Mice with a targeted disruption of the AE2 Cl-/HCO3- exchanger are achlorhydric. The Journal of biological chemistry. 2004;279(29):30531–9. 10.1074/jbc.M403779200 . 15123620

[22] Balestrieri B, Hsu VW, Gilbert H, Leslie CC, Han WK, Bonventre JV, et al Group V secretory phospholipase A2 translocates to the phagosome after zymosan stimulation of mouse peritoneal macrophages and regulates phagocytosis. The Journal of biological chemistry. 2006;281(10):6691–8. 10.1074/jbc.M508314200 16407308 PMC1820836

[23] Balestrieri B, Maekawa A, Xing W, Gelb MH, Katz HR, Arm JP. Group V secretory phospholipase A2 modulates phagosome maturation and regulates the innate immune response against Candida albicans. Journal of immunology. 2009;182(8):4891–8. 10.4049/jimmunol.0803776 19342668 PMC2746418

[24] Hishikawa T, Cheung JY, Yelamarty RV, Knutson DW. Calcium transients during Fc receptor-mediated and nonspecific phagocytosis by murine peritoneal macrophages. The Journal of cell biology. 1991;115(1):59–66. 1918139 10.1083/jcb.115.1.59 PMC2289912

[25] Sato K, Yang XL, Yudate T, Chung JS, Wu J, Luby-Phelps K, et al Dectin-2 is a pattern recognition receptor for fungi that couples with the Fc receptor gamma chain to induce innate immune responses. The Journal of biological chemistry. 2006;281(50):38854–66. 10.1074/jbc.M606542200 . 17050534

[26] McGreal EP, Rosas M, Brown GD, Zamze S, Wong SY, Gordon S, et al The carbohydrate-recognition domain of Dectin-2 is a C-type lectin with specificity for high mannose. Glycobiology. 2006;16(5):422–30. 10.1093/glycob/cwj077 . 16423983

[27] Gawenis LR, Bradford EM, Alper SL, Prasad V, Shull GE. AE2 Cl-/HCO3- exchanger is required for normal cAMP-stimulated anion secretion in murine proximal colon. American journal of physiology Gastrointestinal and liver physiology. 2010;298(4):G493–503. 10.1152/ajpgi.00178.2009 20110461 PMC2853300

[28] Concepcion AR, Salas JT, Sarvide S, Saez E, Ferrer A, Lopez M, et al Anion exchanger 2 is critical for CD8(+) T cells to maintain pHi homeostasis and modulate immune responses. European journal of immunology. 2014;44(5):1341–51. 10.1002/eji.201344218 . 24515893

[29] Frische S, Zolotarev AS, Kim YH, Praetorius J, Alper S, Nielsen S, et al AE2 isoforms in rat kidney: immunohistochemical localization and regulation in response to chronic NH4Cl loading. American journal of physiology Renal physiology. 2004;286(6):F1163–70. 10.1152/ajprenal.00409.2003 . 14749257

[30] Recalde S, Muruzabal F, Looije N, Kunne C, Burrell MA, Saez E, et al Inefficient chronic activation of parietal cells in Ae2a,b(-/-) mice. The American journal of pathology. 2006;169(1):165–76. 10.2353/ajpath.2006.051096 16816370 PMC1698767

[31] Aliprantis AO, Yang RB, Mark MR, Suggett S, Devaux B, Radolf JD, et al Cell activation and apoptosis by bacterial lipoproteins through toll-like receptor-2. Science. 1999;285(5428):736–9. . 10426996 10.1126/science.285.5428.736

[32] van Vugt MJ, Kleijmeer MJ, Keler T, Zeelenberg I, van Dijk MA, Leusen JH, et al The FcgammaRIa (CD64) ligand binding chain triggers major histocompatibility complex class II antigen presentation independently of its associated FcR gamma-chain. Blood. 1999;94(2):808–17. . 10397749

[33] Bi L, Gojestani S, Wu W, Hsu YM, Zhu J, Ariizumi K, et al CARD9 mediates dectin-2-induced IkappaBalpha kinase ubiquitination leading to activation of NF-kappaB in response to stimulation by the hyphal form of Candida albicans. The Journal of biological chemistry. 2010;285(34):25969–77. 10.1074/jbc.M110.131300 20538615 PMC2923990

[34] Kerscher B, Willment JA, Brown GD. The Dectin-2 family of C-type lectin-like receptors: an update. International immunology. 2013;25(5):271–7. 10.1093/intimm/dxt006 23606632 PMC3631001

[35] Means TK, Mylonakis E, Tampakakis E, Colvin RA, Seung E, Puckett L, et al Evolutionarily conserved recognition and innate immunity to fungal pathogens by the scavenger receptors SCARF1 and CD36. The Journal of experimental medicine. 2009;206(3):637–53. 10.1084/jem.20082109 19237602 PMC2699123

[36] Lecanda J, Urtasun R, Medina JF. Molecular cloning and genomic organization of the mouse AE2 anion exchanger gene. Biochemical and biophysical research communications. 2000;276(1):117–24. 10.1006/bbrc.2000.3439 . 11006093

[37] Medina JF, Lecanda J, Acin A, Ciesielczyk P, Prieto J. Tissue-specific N-terminal isoforms from overlapping alternate promoters of the human AE2 anion exchanger gene. Biochemical and biophysical research communications. 2000;267(1):228–35. 10.1006/bbrc.1999.1951 . 10623603

[38] Khan NS, Kasperkovitz PV, Timmons AK, Mansour MK, Tam JM, Seward MW, et al Dectin-1 Controls TLR9 Trafficking to Phagosomes Containing beta-1,3 Glucan. Journal of immunology. 2016;196(5):2249–61. 10.4049/jimmunol.1401545 26829985 PMC4761466

[39] Alexopoulou L, Holt AC, Medzhitov R, Flavell RA. Recognition of double-stranded RNA and activation of NF-kappaB by Toll-like receptor 3. Nature. 2001;413(6857):732–8. 10.1038/35099560 . 11607032

[40] Aliprantis AO, Ueki Y, Sulyanto R, Park A, Sigrist KS, Sharma SM, et al NFATc1 in mice represses osteoprotegerin during osteoclastogenesis and dissociates systemic osteopenia from inflammation in cherubism. The Journal of clinical investigation. 2008;118(11):3775–89. 10.1172/JCI35711 18846253 PMC2564610

[41] Wongchana W, Lawlor RG, Osborne BA, Palaga T. Impact of Notch1 Deletion in Macrophages on Proinflammatory Cytokine Production and the Outcome of Experimental Autoimmune Encephalomyelitis. Journal of immunology. 2015;195(11):5337–46. 10.4049/jimmunol.1401770 26503951 PMC4691704

[42] Akiyama TE, Sakai S, Lambert G, Nicol CJ, Matsusue K, Pimprale S, et al Conditional disruption of the peroxisome proliferator-activated receptor gamma gene in mice results in lowered expression of ABCA1, ABCG1, and apoE in macrophages and reduced cholesterol efflux. Molecular and cellular biology. 2002;22(8):2607–19. 11909955 10.1128/MCB.22.8.2607-2619.2002 PMC133709

[43] Kimura M, Toth LA, Agostini H, Cady AB, Majde JA, Krueger JM. Comparison of acute phase responses induced in rabbits by lipopolysaccharide and double-stranded RNA. The American journal of physiology. 1994;267(6 Pt 2):R1596–605. . 7810770 10.1152/ajpregu.1994.267.6.R1596

[44] Mansour MK, Tam JM, Khan NS, Seward M, Davids PJ, Puranam S, et al Dectin-1 activation controls maturation of beta-1,3-glucan-containing phagosomes. The Journal of biological chemistry. 2013;288(22):16043–54. 10.1074/jbc.M113.473223 23609446 PMC3668760

[45] Ferwerda B, Ferwerda G, Plantinga TS, Willment JA, van Spriel AB, Venselaar H, et al Human dectin-1 deficiency and mucocutaneous fungal infections. The New England journal of medicine. 2009;361(18):1760–7. 10.1056/NEJMoa0901053 19864674 PMC2773015

[46] Goodridge HS, Wolf AJ, Underhill DM. Beta-glucan recognition by the innate immune system. Immunological reviews. 2009;230(1):38–50. 10.1111/j.1600-065X.2009.00793.x . 19594628 PMC6618291

[47] Hise AG, Tomalka J, Ganesan S, Patel K, Hall BA, Brown GD, et al An essential role for the NLRP3 inflammasome in host defense against the human fungal pathogen Candida albicans. Cell host & microbe. 2009;5(5):487–97. 10.1016/j.chom.2009.05.002 19454352 PMC2824856

[48] Davis JT, Gale PA, Okunola OA, Prados P, Iglesias-Sanchez JC, Torroba T, et al Using small molecules to facilitate exchange of bicarbonate and chloride anions across liposomal membranes. Nature chemistry. 2009;1(2):138–44. 10.1038/nchem.178 . 21378827

[49] Davis JT, Okunola O, Quesada R. Recent advances in the transmembrane transport of anions. Chemical Society reviews. 2010;39(10):3843–62. 10.1039/b926164h . 20820462

